# Evaluation of operation outcomes in patients with degenerative lumbar scoliosis

**Published:** 2012-11

**Authors:** Majid Reza Farrokhi, Mohammad Jamali

**Affiliations:** ^*a*^Shiraz Neurosciences Research Center, Neurosurgery Department, Shiraz University of Medical Sciences, Shiraz, Iran.

## Abstract

**Background:**

Due to the increase of life expectancy, the prevalence of degenerative spine diseases such as adult degenerative lumbar scoliosis (ADLS) has been increasingly more than before which is required appropriate control and management. Therefore, the present study was aimed to survey the result of surgery in ADLS patients in Shiraz (Iran).

**Methods:**

This is a preliminary report of 30 patients with ADLS who underwent pedicular screw fixation, posterolateral fusion, posterior decompression and correction of coronal plane deformity operation according to surgical indications in the Chamran and Kowsar hospitals during 2009-2011. The patients were followed up at 1, 6 and 12 months post operation. Radiologic changes were evaluated and the Oswestry low back pain disability (OLBP) scale and visual analogue scale (VAS) were used to evaluate functional and pain improvement, respectively. Data were analyzed using SPSS software version 15. We used Wilcoxon signed-rank test to compare the parameters of pain and LBP scale.

**Results:**

Primary analysis showed that 42.9% of operated patients were in 50-60 years age group. 71% of the patients were female and 29% were male. Prevalence of LBP from radicular pain among the patients was 95.2%. There was a significant difference between pre-operation and post-operation VAS and Oswestry LBP scale (P less than 0.001).

**Conclusions:**

Our findings showed that posterior decompression combined with pedicular fixation, posterolateral fusion and correction of coronal plane deformity seems to be a suitable method for the relief of pain and improvement of function in ADLS patients. Only decompression can relieve low back pain but for the relief of radicular pain and correction of deformity, fixation and fusion are recommended.

**Keywords:**

Scoliosis, Degenerative, Low back pain, Adult lumbar, Radicular pain


					Volume 4, Suppl. 1 Nov 2012
Publisher: Kermanshah University of Medical Sciences
URL: http://www.jivresearch.org
Copyright:
Congress of Iranian Neurosurgeons, 14-16 November 2012, Kermanshah, Iran
Paper No. 7
Evaluation of operation outcomes in patients with degenerative
lumbar scoliosis
Majid Reza Farrokhi a,*
, Mohammad Jamali a
a
Shiraz Neurosciences Research Center, Neurosurgery Department, Shiraz University of Medical Sciences, Shiraz, Iran.
Abstract:
Background: Due to the increase of life expectancy, the prevalence of degenerative spine diseases such as adult
degenerative lumbar scoliosis (ADLS) has been increasingly more than before which is required appropriate control
and management. Therefore, the present study was aimed to survey the result of surgery in ADLS patients in Shiraz
(Iran).
Methods: This is a preliminary report of 30 patients with ADLS who underwent pedicular screw fixation,
posterolateral fusion, posterior decompression and correction of coronal plane deformity operation according to
surgical indications in the Chamran and Kowsar hospitals during 2009-2011. The patients were followed up at 1, 6
and 12 months post operation. Radiologic changes were evaluated and the Oswestry low back pain disability
(OLBP) scale and visual analogue scale (VAS) were used to evaluate functional and pain improvement, respectively.
Data were analyzed using SPSS software version 15. We used Wilcoxon signed-rank test to compare the
parameters of pain and LBP scale.
Results: Primary analysis showed that 42.9% of operated patients were in 50-60 years age group. 71% of the
patients were female and 29% were male. Prevalence of LBP from radicular pain among the patients was 95.2%.
There was a significant difference between pre-operation and post-operation VAS and Oswestry LBP scale (P less
than 0.001).
Conclusions: Our findings showed that posterior decompression combined with pedicular fixation, posterolateral
fusion and correction of coronal plane deformity seems to be a suitable method for the relief of pain and
improvement of function in ADLS patients. Only decompression can relieve low back pain but for the relief of
radicular pain and correction of deformity, fixation and fusion are recommended.
Keywords:
Scoliosis, Degenerative, Low back pain, Adult lumbar, Radicular pain
* Corresponding Author at:
Prof. Majid Reza Farrokhi: Professor of Neurosurgery, Shiraz Neurosciences Research Center, Chamran Hospital, Chamran Boulevard, Shiraz, Iran.
Mob: 09171114472, Tel: +98711-6234508, Fax: +98711-6234508, E-mail: farokhim@sums.ac.ir, farrokhi_drmr@yahoo.com, (Farrokhi MR.).

				

